# Correlation of vascular and fluid‐related parameters in neovascular age‐related macular degeneration using deep learning

**DOI:** 10.1111/aos.15219

**Published:** 2022-08-01

**Authors:** Markus Schranz, Reinhard Told, Valentin Hacker, Gregor S. Reiter, Adrian Reumueller, Wolf‐Dieter Vogl, Hrvoje Bogunovic, Stefan Sacu, Ursula Schmidt‐Erfurth, Philipp K. Roberts

**Affiliations:** ^1^ Vienna Clinical Trial Center (VTC), Department of Ophthalmology and Optometry Medical University of Vienna Vienna Austria; ^2^ Department of Ophthalmology and Optometry Medical University of Vienna Vienna Austria; ^3^ OPTIMA, Christian Doppler Laboratory, Department of Ophthalmology and Optometry Medical University of Vienna Vienna Austria

## Abstract

**Purpose:**

To identify correlations between the vascular characteristics of macular neovascularization (MNV) obtained by optical coherence tomography angiography (OCTA) and distinct retinal fluid volumes in neovascular age‐related macular degeneration (nAMD).

**Methods:**

In this prospective interventional study, 54 patients with treatment‐naïve type 1 or 2 nAMD were included and treated with intravitreal aflibercept. At baseline and month 1, each patient underwent a SD‐OCT volume scan and volumetric flow scan using a swept‐source OCTA. A deep learning algorithm was used to automatically detect and quantify fluid in OCT scans. Angio Tool, a National Cancer Institute algorithm, was used to skeletonize MNV properties and quantify lesion size (LS), vessel area (VA), vessel density (VD), total number of endpoints (TNE), total number of junctions (TNJ), junction density (JD), total vessel length (TVL), average vessel length (AVL) and mean‐e‐lacunarity (MEL). Subsequently, linear regression models were used to investigate a correlation between OCTA parameters and fluid quantifications.

**Results:**

The median amount of fluid within the central 6‐mm EDTRS ring was 173.7 nl at baseline, consisting of 156.6 nl of subretinal fluid (SRF) and 2.3 nl of intraretinal fluid (IRF). Fluid decreased significantly in all compartments to 1.76 nl (SRF) and 0.64 nl (IRF).

The investigated MNV parameters did not change significantly after the first treatment.

There was no significant correlation between MNV parameters and relative fluid decrease after anti‐VEGF treatment. Baseline fluid correlated statistically significant but weakly with TNE (*p* = 0.002, *R* ^2^ = 0.17), SRF with TVL (*p* = 0.04, *R* ^2^ = 0.08), VD (*p* = 0.046, *R* ^2^ = 0.08), TNE (*p* = 0.001, *R* ^2^ = 0.20) and LS (*p* = 0.033, *R* ^2^ = 0.09). IRF correlated with VA (*p* = 0.042, *R* ^2^ = 0.08).The amount of IRF at month 1 correlated significantly but weakly with VD (*p* = 0.036, *R* ^2^ = 0.08), JD (*p* = 0.019, *R* ^2^ = 0.10) and MEL (*p* = 0.005, *R* ^2^ = 0.14).

**Conclusion:**

Macular neovascularization parameters at baseline and month 1 played only a minor role in the exudation process in nAMD. None of the MNV parameters were correlated with the treatment response.

## 1 INTRODUCTION

Advances in artificial intelligence (AI) offer new methods to analyse and evaluate the impact of biomarkers on visual acuity and treatment response in retinal diseases such as age‐related macular degeneration (AMD) (Bogunovic et al., 2017; Schmidt‐Erfurth et al., 2020). 10%–15% of patients were diagnosed with AMD develop macular neovascularization (MNV), which if not treated early can cause rapid and irreversible vision loss, due to exudation in the form of retinal fluid (RF), bleeding and scar‐formation (Schmidt‐Erfurth et al., 2014; Wong et al., 2014).

Deep learning algorithms are used to automatically detect, localize and quantify RF in optical coherence tomography (OCT) volume scans, which allow exact tracking of fluid changes under therapy and give a more precise insight into disease activity (Schlegl et al., 2018).

Recent studies have shown that subretinal (SRF) or intraretinal fluid (IRF) affect visual acuity differently. For example, SRF has a lesser negative impact on visual acuity than IRF (Guymer et al., 2019; Reiter et al., 2021). Furthermore, it has been shown that especially in neovascular AMD (nAMD) the volume of IRF and SRF do not correlate with central subfield thickness (CST) (Pawloff et al., 2022).

This highlights the importance of distinct fluid quantification and localization and shows benefits of deep learning–based imaging biomarkers over other biomarker such as CST.

Besides RF, the detection and quantification of MNV membranes by OCT angiography (OCTA) is of increasing interest.

By delivering a detailed visualization of microvascular morphology within the MNV membrane, new biomarkers can be identified (Bae et al., 2019; Cicinelli et al., 2019; Liang et al., 2016; Novais et al., 2016; Told et al., 2019). However, all of the studies published in this field are based on surrogates such as presence of fluid or retinal thickness (Coscas et al., 2018; Faatz et al., 2019; Roberts et al., 2017; Told, Reiter, Schranz, et al., 2020; Told, Reiter, Mittermüller, et al., 2020).

Currently, the choice of the appropriate treatment regimens is based mostly on the presence and location of fluid in OCT. The two most commonly used anti‐vascular endothelial growth factor (anti‐VEGF) treatment regimen, treat and extend and pro‐re‐nata, are both time and cost intensive and might result in over or under treatment, respectively (Hatz & Prünte, 2017).

It remains to be elucidated whether OCTA‐related MNV biomarkers are correlated with fluid volume and dynamics and hence may be used to predict individual disease severity at baseline as well as disease activity. Personalized treatment based on baseline MNV parameters could potentially reduce patient visits and associated costs and improve patient management and comfort.

The aim of this study was to perform a comprehensive analysis of accurate fluid volumes in standard structural OCT and the vascular characteristics of MNV imaged by OCTA. We examined treatment‐naïve eyes at baseline and 30 days after the first anti‐VEGF injection using aflibercept. Since the biggest reduction is to be expected in this time period (Michl et al., 2020), we were interested in possible correlations between the change in MNV parameters and the change in fluid after the first treatment.

## 2 METHODS

The present prospective longitudinal study was conducted at the Department of Ophthalmology and Optometry of the Medical University of Vienna (Vienna, Austria). The study was approved by the local ethics committee (Ethikkommission der Medizinischen Universität Wien; Approval number: EK1844/2015) and adhered to the tenets of the Declaration of Helsinki. Every patient gave written informed consent prior to study inclusion.

Fifty‐four eyes of 54 consecutive patients with treatment‐naïve neovascular AMD (nAMD) were included between May 2017 and May 2019.

Inclusion criteria were age 50 years or older, clear ocular media, good fixation ability and best corrected distance visual acuity (BCVA) between 0.097 and 1.20 logMAR (20/25–20/320 Snellen equivalent).

Exclusion criteria were myocardial stroke and/or brain insult within 12 months prior to baseline, uveitis, glaucoma, type 3 MNV and choroidal neovascularization (CNV) secondary to diseases other than AMD.

Each patient underwent following examinations at baseline and 1 month (M1) after the first anti‐vascular endothelial growth factor (anti‐VEGF) intravitreal injection using aflibercept given at baseline: BCVA testing using Early Treatment Diabetic Retinopathy Study charts (ETDRS), swept‐source optical coherence tomography angiography (SS‐OCTA) using the PlexElite device (Carl Zeiss Meditec Inc., Dublin, CA, USA), slitlamp‐biomicroscopy, dilated fundus examination, fluorescein and indocyanine green angiography (FLA, ICGA) at baseline and spectral domain OCT (SD‐OCT) using the HRA + OCT device (Spectralis, Heidelberg Engineering, Germany).

An image quality above 30 for the SD‐OCT and a signal‐to‐noise ratio of at least 7 for the SS‐OCT was mandatory, if not achieved the scan was repeated or the patient excluded.

Macular neovascularization classification was made by two retinologists (G.R. and A.R.) according to the criteria based on new consensus guidelines on MNV types using OCT and OCTA (Spaide et al., 2020). In type I, the MNV membrane is located between the retinal pigment epithelium (RPE) and Bruch's membrane; in type 2, the lesion is located above the RPE; and in a mixed type I and 2, type MNV is found in both compartments.

Disagreements were evaluated by a third retinologist (S.S) and a consensus grading was made.

## 3 IMAGING AND IMAGE ANALYSIS

### 3.1 OCT volumetric scans

A fovea‐centred high‐resolution 97‐line raster with an image averaging of 15 images per line was performed in the in‐built follow‐up mode, based on image registration, using the HRA + OCT device.

**FIGURE 1:**
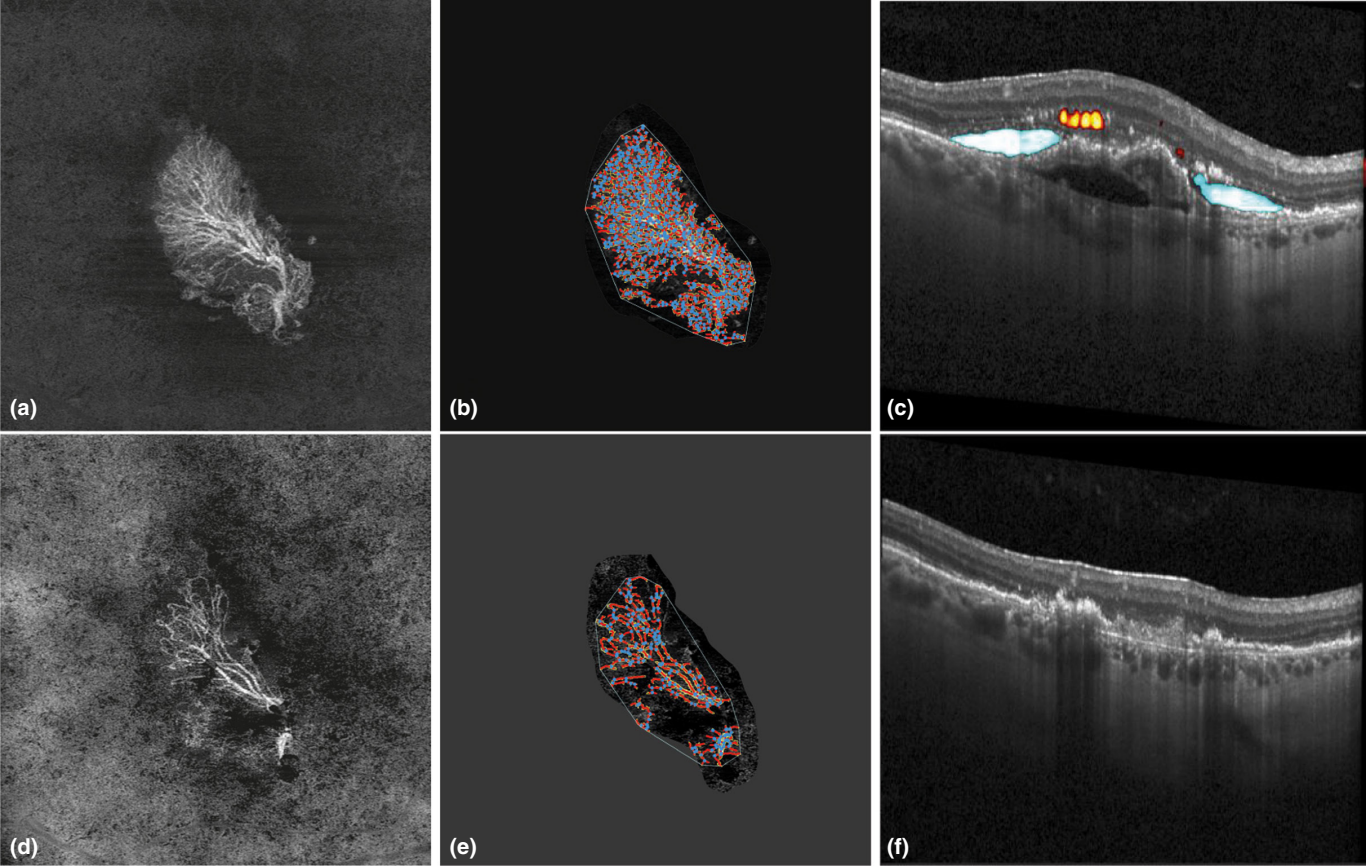
Image (a) and (d) show the en‐face scan of a type I MNV membrane at baseline (a) and 30 days (d) after the first anti‐VEGF intravitreal injection. Images (b) and (e) show skeletonized visualizations of the MNV membrane using the AngioTool software (red lines represent vessels, and blue dots junctions). Images (c) and (f) show the B scans with deep learning–based fluid detection at the levels of the red lines in images (a) and (d). Automatically detected intraretinal fluid is in orange‐yellow colour and subretinal fluid in blue.

Raw data were exported as vol files for further processing. The foveal centre was annotated manually by a retinologist (M.S) in OCT volume scans using the in‐house software OPTIMUS (Medical University Vienna; Vienna, Austria) to provide the centre point for the EDTRS grid. In most eyes, the foveal contour was preserved; hence, the fovea was easily identified. In eyes with distortion of the foveal contour, due to the presence of IRF or SRF, the fovea was identified by the absence of the inner retinal layers. Subsequently, automated layer segmentation, fluid detection and quantification were performed using a convolutional neuronal network (CNN). The architecture of this deep learning algorithm has been described and extensively validated previously (Schlegl et al., 2018; Schmidt‐Erfurth et al., 2020). From the segmented OCT scan, fluid volume in the central 6‐mm EDTRS disk of the macula was calculated (see Figure 1c,f).

### 3.2 OCTA scans

A fovea‐centred flow volume scan was acquired using the PlexElite 9000 1060 nm SS‐OCTA (Carl Zeiss Meditec Inc., Dublin, CA, USA). The acquisition parameters of this scan were predefined to include a 6 × 6 mm scanning area with 500 × 500 A‐scans (scan rate: 100 000, axial resolution: 6.3 μm). Every B‐scan was repeated twice.

The so‐called RPE‐RPE fit (for type I MNV) or outer retina to choriocapillaris (for type 2 MNV) slab were used to display MNV membranes in the en‐face image. If necessary, segmentation was adjusted manually to ensure that the orthogonal visualization only contains flow signal from the MNV. The in‐built projection artefact removal of the PlexElite was used to remove flow artefacts to improve contrast and quality (Zhang et al., 2017). Subsequently, the en‐face image was exported as TIF image with a resolution of 1024 × 1024 pixels for further image processing and analysis (see Figure 1a).

Images were processed with the following series of steps: (1) Import TIF image into ImageJ (V1.53g) for contouring MNV membrane area and to cut out the surrounding background. Save the resulting image as a TIF. (2) Load image in AngioTool (0.6a, National Cancer Institute, USA) software. (3) Adapt following parameters individually for each image to obtain optimal vessel delineation: threshold, vessel thickness, removal of small particles, image resolution (1024 × 1024 pixels), image size (6 × 6 mm). (4) Start semiautomatic analysis and export settings and results to an XLS file (Zudaire et al., 2011).

The resulting parameters and corresponding abbreviations to this analysis are listed in Table 1, and an example can be seen in Figure 1b,e.

**TABLE 1:** Obtained vessel parameters and their definition as provided by AngioTool

Analyzed macular neovascular membrane parameters	
Parameter	Definition
Lesion size (LS)	The area covered by the macular neovascular membrane (mm^2^)
Vessel area (VA)	The total area of OCTA flow information within the lesion (mm^2^)
Vessel density (VD)	The percentage of flow information within the total lesion (%)
Total vessel length (TVL)	The sum of the length of vessels between two junctions or endpoints (mm)
Average vessel length (AVL)	The mean distance between two junctions or endpoints of all vessels (mm)
Total number of junctions (TNJ)	The total number of vessel junctions within the lesion
Junction density (JD)	The total number of junctions per unit area
Number of vessel endpoints (NE)	The total number of vessel dead ends
Mean‐e‐lacunarity (MEL)	A measure of MNV homogeneity (higher values correspond to a more inhomogeneous lesion)

### 3.3 Statistical testing

For statistical testing, SPSS Statistic (Version 25.0; Armonk, NY: IBM Corp.) and RStudio (Version 1.2.1335© 2009–2019 RStudio, Inc.) were used. Evaluation for normal distribution was done using boxplots and the Shapiro–Wilk test. Normal distributed data are reported as mean ± standard deviation. ANOVA was used to compare means between independent and dependent groups, and Šídák's multiple comparisons test was used to correct for multiple testing.

Non‐normally distributed data are reported as median and interquartile range (1st and 3rd quartile), the Friedman test was used to compare dependent groups and Dunn's test was used to correct for multiple testing. Linear regression analyses and mixed models were used for analyses of association between fluid volumes and MNV microstructure characteristics. Fluid volume and MNV parameters were normalized by logarithmizing prior to regression analysis. Variance inflation factor (VIF) analysis was used to quantify and clear for multicollinearity in the mixed models. The level of significance was set to α = 0.05.

## 4 RESULTS

In total, 54 eyes of 54 patients (36 female: 67%) with treatment‐naïve nAMD were included. The mean age at treatment start was 76.9 ± 6.6 years, with no significant difference between male and female patients considering age (*p* = 0.86). In total, 35 (65%) eyes showed type I, 4 (7%) type 2 and 15 (28%) mixed type I and 2 MNV. Mean visual acuity at baseline was 0.28 ± 0.59 logMAR. All eyes surpassed the required minimal image quality in OCT and OCTA.

### 4.1 Fluid volumes and MNV parameters at baseline and month 1

The amount of fluid was not normally distributed. The median amount of total RF within the central 6 mm was 173.69 nL (84.74; 614.49) at baseline. The median amount of SRF was 156.61 nL (60.31; 435.83), and the median amount of IRF was 2.34 nl (0.68; 22.34). At M1, total RF, SRF and IRF decreased significantly to a median of 5.02 nl (0.96; 18.97), 1.76 nl (0.05; 14.63) and 0.64 nl (0.17; 2.08), respectively (all *p* < 0.001).

No significant changes in the MNV parameters between baseline and M1 could be observed. A detailed overview of RF and MNV parameters can be seen in Table 2.

**TABLE 2:** Median (1st quartile, 3rd quartile) amount of fluid in the different retinal compartments and median (1st quartile, 3rd quartile) values of the MNV membrane parameters at baseline and follow up (FUP: 30 days after the first anti‐VEGF intravitreal injection)

Fluid and macula neovascular membrane findings at baseline and month 1			
	Baseline	Follow up	*p*‐Value
Total fluid (nl)	173.69 (084.74; 614.49)	005.02 (000.96; 018.97)	**<0.001**
SRF (nl)	156.61 (060.31; 435.83)	001.76 (000.05; 014.63)	**<0.001**
IRF (nl)	002.34 (000.68; 022.34)	000.64 (000.17; 002.08)	**<0.001**
Vessel area (mm^2^)	000.68 (000.25; 001.32)	000.43 (000.19; 001.00)	>0.999
Vessel density (%)	033.21 (028.17; 041.24)	028.15 (022.87; 035.81)	>0.999
Total number of junctions (*n*)	157.00 (070.25; 413.00)	140.50 (044.00; 295.00)	>0.999
Junctions density	108.30 (058.70; 135.19)	083.04 (052.15; 136.17)	>0.999
Total vessel length (mm)	023.65 (010.22; 045.90)	017.64 (006.92; 036.18)	>0.999
Average vessel length (mm)	008.57 (002.32; 020.60)	004.56 (002.13; 012.26)	>0.999
Total number of endpoints (*n*)	078.50 (041.00; 164.50)	089.00 (030.00; 157.50)	>0.999
Mean‐e‐lacunarity (au)	000.27 (000.17; 000.34)	000.27 (000.20; 000.43)	>0.999

*Note*: The *p*‐value indicates significance in difference between the two timepoints, Friedman test was used to compare medians between baseline and follow up, Dunn's test was used to correct for multiple comparison. Statistically significant values in bold.

### 4.2 Correlation between MNV features and RF volume at baseline

Macular neovascularization properties at baseline in treatment‐naïve eyes and their effect on the amount of baseline RF were analysed using a uni‐ and multivariable mixed‐effects model. Total RF volume at baseline correlated statistically significantly but weakly with total number of endpoints (TNE) (*R* ^2^ = 0.17, *p* = 0.002). SRF volume at baseline correlated significantly but weakly with vessel density (VD) (*R* ^2^ = 0.08, *p* = 0.046), total vessel length (TVL) (*R* ^2^ = 0.09, *p* = 0.04), TNE (*R* ^2^ = 0.2, *p* = 0.0008) and lesion size (LS) (*R* ^2^ = 0.09, *p* = 0.033). IRF volume correlated significantly but weakly with vessel area (VA) (*R* ^2^ = 0.08, *p* = 0.042). For more details, see Table 3.

**TABLE 3:** Univariable regression analysis between macular neovascularmeembrane (MNV) parameters at baseline and retinal fluid volume at baseline (MNV parameter values and fluid volume are logarithmized, log‐log model), († a coefficient with an absolute value <0.01)

Univariable regression analysis of baseline fluid and baseline macular neovascular membrane parameters									
	Total retinal fluid volume			Subretinal fluid volume			Intraretinal fluid volume		
	Coefficient	*p*‐Value	Multiple *R* ^2^	Coefficient	*p*‐Value	Multiple *R* ^2^	Coefficient	*p*‐Value	Multiple *R* ^2^
MNV‐property (characteristic)									
Vessel area	0.24	0.119	0.05	0.25	0.119	0.05	**1.08**	**0.042**	**0.08**
Vessel density	−0.01	0.162	0.04	**−0.01**	**0.046**	**0.08**	0.00†	0.173	0.04
Total number of junctions	0.24	0.082	0.06	0.26	0.062	0.07	0.84	0.079	0.06
Junction density	0.00†	0.879	0.00	0.00†	0.919	0.00	0.01	0.356	0.02
Total vessel length (mm)	0.30	0.055	0.07	**0.33**	**0.040**	**0.08**	1.06	0.055	0.07
Average vessel length	−0.01	0.152	0.57	−0.01	0.221	0.03	0.01	0.346	0.02
Total number of endpoints	**0.51**	**0.002**	**0.17**	**0.57**	**0.001**	**0.20**	0.71	0.236	0.03
Mean E Lacunarity	−0.29	0.298	0.02	−0.17	0.546	0.01	−1.64	0.089	0.05
Lesion size	0.05	0.052	0.07	**0.05**	**0.033**	**0.09**	1.02	0.078	0.06

Statistically significant values in bold.

Multivariable analyses showed a statistically significant but weak correlation for RF volume (*R* ^2^ = 0.205; *p* = 0.0039) and SRF volume (*R* ^2^ = 0.228; *p* = 0.002), which included VD, average vessel length (AVL), TNE and mean‐e‐lacunarity (MEL). For more details, see Table 4.

**TABLE 4:** Multivariable regression analysis between macular neovascularization (MNV) parameters at baseline and retinal fluid volume at baseline (MNV parameter values and fluid volume are logarithmized, log‐log model) (*p‐value < 0.001)

Multivariable regression analysis of baseline fluid and baseline macular neovascular membrane parameters						
	Coefficient	*p*‐Value	Variance inflation factor	Residual standard error model	Adjusted *R*‐squared Model	*p*‐Value model
Total retinal fluid						
Vessel density	0.63	0.450	3.9	**0.516**	**0.205**	**0.004**
Average vessel length	**−0.37**	**0.018**	**1.8**			
Total number of endpoints	**0.70**	0.000*	**1.5**			
Mean E Lacunarity	0.16	0.731	3.4			
Subretinal fluid						
Vessel density	0.35	0.670	3.9	**0.528**	**0.228**	**0.002**
Average vessel length	**−0.31**	**0.047**	**1.8**			
Total number of endpoints	**0.75**	0.000*	**1.3**			
Mean E Lacunarity	0.22	0.650	3.4			
Intraretinal fluid						
Vessel Density	−3.17	0.300	3.9	1.93	0.054	0.154
Average vessel length	0.42	0.460	1.8			
Total number of endpoints	0.22	0.740	1.3			
Mean E Lacunarity	−3.14	0.073	3.4			

Statistically significant values in bold.

### 4.3 Correlation between MNV parameters and the relative decrease of RF after anti‐VEGF treatment

To find correlations between baseline MNV parameters and relative fluid decrease (baseline to M1), a univariable and multivariable regression model was used. None of the investigated MNV characteristics were correlated significantly with the decrease of any RF compartment. For more details, see Table 5.

**TABLE 5:** Univariable regression analysis between normalized macular neovascular (MNV) parameters at baseline and the relative decrease of retinal fluid after the first treatment (MNV parameter values are logarithmized, log‐linear model) († a coefficient with an absolute value < 0.01)

Univariable regression analysis of macular neovascular membrane parameters at baseline and relative fluid decrease									
	Decrease Total retinal fluid			Decrease Subretinal fluid			Decrease Intraretinal fluid		
	Coefficient	*p*‐Value	Multiple *R* ^2^	Coefficient	*p*‐Value	Multiple *R* ^2^	Coefficient	*p*‐Value	Multiple *R* ^2^
MNV‐property (characteristic)									
Vessel Area	−0.01	0.901	0.00	−0.02	0.703	0.00	0.46	0.695	0.00
Vessel Density	0.00†	0.460	0.01	0.00†	0.460	0.01	0.05	0.355	0.02
Total number of junctions	−0.02	0.762	0.00	−0.02	0.762	0.00	0.50	0.636	0.01
Junctions density	0.00	0.206	0.03	0.00†	0.206	0.03	0.02	0.228	0.03
Total vessel length (mm)	−0.02	0.822	0.00	−0.02	0.822	0.00	0.42	0.735	0.00
Average vessel length	0.00†	0.695	0.00	0.00†	0.695	0.00	0.00	0.816	0.00
Total number of endpoints	−0.03	0.716	0.00	−0.03	0.716	0.00	0.12	0.929	0.00
Mean E Lacunarity	0.10	0.412	0.01	0.10	0.412	0.01	−1.10	0.610	0.00
Lesion size	0.02	0.824	0.00	0.02	0.824	0.00	0.10	0.937	0.00

Statistically significant values in bold.

### 4.4 Correlation between MNV parameters and residual fluid at M1

To assess correlations between MNV parameters in treated eyes and the amount of RF, we analysed MNV parameters at M1 with the amount of fluid measured at the same time point.

In a univariable analysis, the amount of RF at day 30 correlated significantly but weakly with VA (c = 0.54, *R* ^2^ = 0.11, *p* = 0.016), VD (c = 1.32, *R* ^2^ = 0.10, *p* = 0.019), total number of junctions (TNJ) (c = 0.49, *R* ^2^ = 0.12, *p* = 0.010), junction density (JD) (c = 1.14, *R* ^2^ = 0.14, *p* = 0.005), TVL (c = 0.53, *R* ^2^ = 0.10, *p* = 0.023), average vessel length (AVL) (c = 0.48, *R* ^2^ = 0.10, *p* = 0.018) and MEL (c = −0.90, *R* ^2^ = 0.10, *p* = 0.018). There was no significant correlation between the MNV parameters and SRF. The amount of IRF correlated significantly but weakly with VD (c = 2.81, *R* ^2^ = 0.08, *p* = 0.036), JD (c = 2.27, *R* ^2^ = 0.10, *p* = 0.019) and MEL (c = −2.40, *R* ^2^ = 0.14, *p* = 0.005). For more details, see Table 6.

**TABLE 6:** Univariable regression analysis between macular neovascular (MNV) parameters and retinal fluid volume (MNV parameter values and fluid volume are logarithmized, log‐log model), 30 days after the first treatment

Univariable regression analysis of macular neovascular membrane parameters at month 1 and fluid at month 1									
	Total retinal fluid			Subretinal fluid			Intraretinal fluid		
	Coefficient	*p*‐Value	Multiple *R* ^2^	Coefficient	*p*‐Value	Multiple *R* ^2^	Coefficient	*p*‐Value	Multiple *R* ^2^
MNV‐property (characteristic)									
Vessel Area	**0.54**	**0.016**	**0.11**	0.57	0.345	0.02	0.78	0.150	0.04
Vessel density	**1.32**	**0.019**	**0.10**	1.8	0.232	0.03	**2.81**	**0.036**	**0.08**
Total number of junctions	**0.49**	**0.010**	**0.12**	0.35	0.493	0.01	0.74	0.107	0.03
Junctions density	**1.14**	**0.005**	**0.14**	0.01	0.350	0.02	**2.27**	**0.019**	**0.10**
Total vessel length	**0.53**	**0.023**	**0.10**	0.46	0.460	0.01	0.73	0.192	0.03
Average vessel length	**0.48**	**0.018**	**0.10**	0.17	0.756	0.00	0.63	0.194	0.03
Total number of endpoints	0.41	0.097	0.05	0.03	0.966	0.00	0.60	0.305	0.02
Mean E Lacunarity	**−0.90**	**0.018**	**0.10**	−1.06	0.304	0.02	**−2.40**	**0.005**	**0.14**
Lesion size	0.37	0.125	0.05	0.32	0.622	0.01	0.38	0.520	0.01

Statistically significant values in bold.

In a multivariable analysis, we found statistically significant but weak correlations for RF (*R* ^2^ = 0.107; *p* = 0.035) and IRF (*R* ^2^ = 0.11; *p* = 0.034) which included AVL, TNE and MEL. For more details, see Table 7.

**TABLE 7:** Multivariable regression analysis between macular neovascular (MNV) properties and retinal fluid volume (MNV parameter values and fluid volume are logarithmized, log‐log model), 30 days after the first treatment

Multivariable regression analysis of macular neovascular membrane parameters at month 1 and fluid at month 1						
	Coefficient	*p*‐Value	Variance inflation factor	Residual standard error model	Adjusted *R*‐square Model	*p*‐Value model
Total retinal fluid						
Average vessel length	**0.20**	**0.441**	**2.0**	**0.87**	**0.107**	**0.035**
Total number of endpoints	**0.23**	**0.432**	**1.6**			
Mean E Lacunarity	−0.72	0.872	1.3			
Subretinal fluid						
Average vessel length	−0.12	0.894	2.0	2.468	‐0.04	0.788
Total number of endpoints	0.06	0.941	1.6			
Mean E Lacunarity	−1.13	0.340	1.3			
Intraretinal fluid						
Average vessel length	−0.28	0.658	2.0	**2.067**	**0.11**	**0.034**
Total number of endpoints	0.71	0.315	1.6			
Mean E Lacunarity	**−2.66**	**0.073**	**1.3**			

Statistically significant values in bold.

**FIGURE 2:**
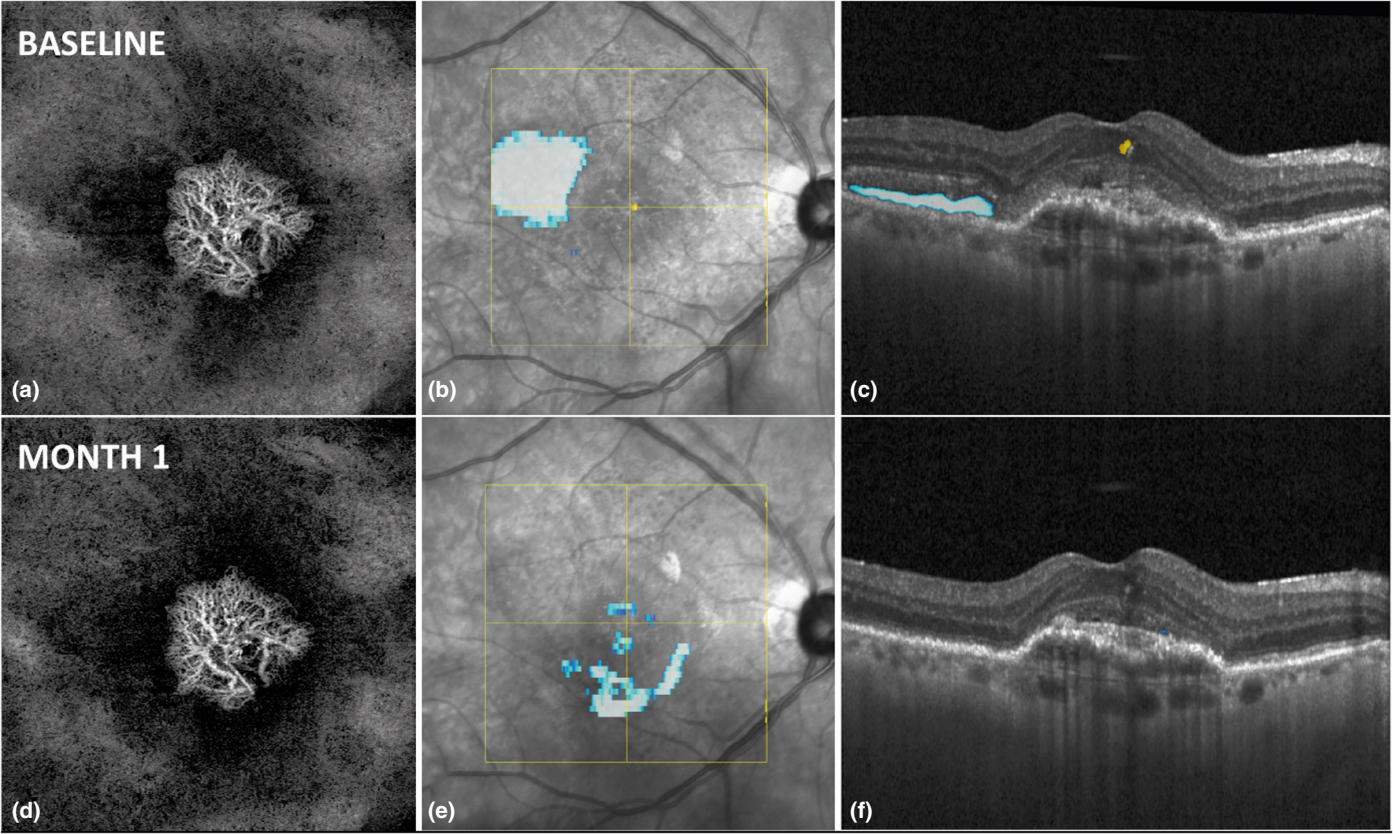
Image (a) shows an OCTA enface image of one study patient at baseline with a mixed type I and 2 MNV and a visual acuity of 20/40. (b) shows the SLO image of the same patient, with AI‐based fluid detection. Blueish areas represent subretinal fluid (SRF), and yellowish areas represent intraretinal fluid (IRF). (c) shows the B scan at the level of the yellow central horizontal line in image (b). Blueish areas represent automatically detected and quantified SRF, and yellowish areas represent IRF. (d), (e) and (f) are the corresponding images 30 days after the first anti‐VEGF treatment.

**FIGURE 3:**
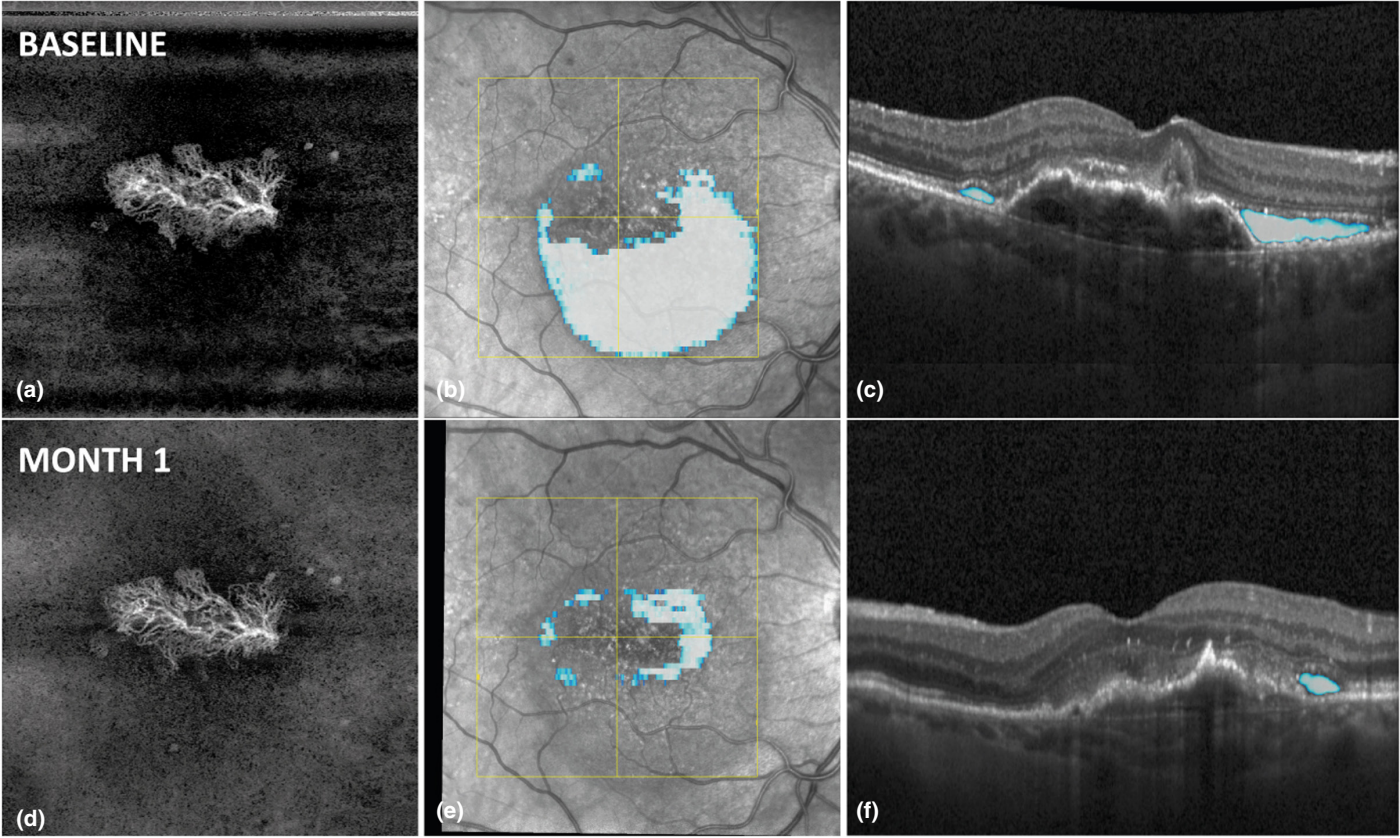
Image (a) shows an OCTA enface image of one study patient at baseline with type I MNV and a visual acuity of 20/40. (b) shows the SLO image of the same patient, with AI‐based fluid detection. Blueish areas represent subretinal fluid (SRF), and yellowish areas represent intraretinal fluid (IRF). (c) shows the B scan at the level of the yellow central horizontal line in image (b). Blueish areas represent automatically detected and quantified SRF, and yellowish areas represent IRF. (d), (e) and (f) are the corresponding images 30 days after the first anti‐VEGF treatment.

Figures 2 and 3 show examples of two patients where fluid decreased significantly however almost no changes in vessel parameters could be seen.

## 5 DISCUSSION

Assessing the influence of MNV membranes on the amount of exudation in nAMD has been a challenging field of research. Many previous studies (Coscas et al., 2018; Faatz et al., 2019; Roberts et al., 2017; Told, Reiter, Schranz, et al., 2020, Told, Reiter, Mittermüller, et al., 2020) lacked distinct parameters, so relative or less precise parameters such as morphological MNV appearance or CRT were used as surrogates.

Recent advances in deep learning algorithms allow analysis of quantifiable MNV parameters together with distinct RF volume to gain new clinical insights in the correlation between MNV structure and disease activity in the form of exudation.

We applied fluid detection and measurement in 54 treatment‐naïve eyes of 54 patients at two time points (baseline, M1) by using a CNN capable of precisely quantifying SRF and IRF in high‐resolution OCT volume scans. MNV properties were analysed based on OCTA images using the semiautomatic open‐source software AngioTool. Those timepoints were intentionally chosen as the largest amount of fluid (SRF, IRF) in nAMD resolves after the first treatment (Michl et al., 2020). Subsequently, any correlation between the fluid decrease and MNV parameters must be visible at this timepoints.

We could show that fluid decreased significantly in all compartments; however, we did not find any clinical correlations between fluid and MNV parameters at the investigated timepoints.

Considering SRF at baseline our result is comparable with the result of the HARBOR trial, where fluid quantification of 1095 patients was performed and a mean of 423 nl of SRF was reported. The amount of IRF at baseline on the other hand was higher in the HARBOR trial with a mean of 142 nl. A reason for this incongruence could be that in this study, patients diagnosed with type 3 MNV, which are associated with higher amounts of IRF, were excluded (Schmidt‐Erfurth et al., 2020).

Analysing univariable correlations between MNV parameters and the amount of RF in treatment‐naïve eyes, we found that there is a statistically significant but weak correlation between SRF volume at baseline and TNE, TVL, LS and VD.

Considering the multivariable analyses after clearing for collinearity (excluded parameters: VA, TVL, TNJ, JD), VD (*p* = 0.67), TNE (*p* < 0.001) and MEL (*p* = 0.65) were positively associated with the amount of SRF at baseline, whereas AVL (*p* < 0.05) was negatively associated. The model reached statistically significance (*p* = 0.002) with an adjusted *R*‐squared value of 0.23, showing a weak correlation.

Recent work by Told, Reiter, Schranz, et al., 2020; Told, Reiter, Mittermüller, et al., 2020. described correlations between retinal thickness in OCT and TVL, TNJ and VA at baseline. However, this paper was based on CFT and not distinct fluid volumetric compartments and did not take the TNE into account, which showed in our univariable as well as in the multivariable analysis a statistically significant association. Empirically, it is not surprising that higher amounts of endpoints are the result of increased vessel sprouting and therefore a sign of MNV growth activity, but though statistically significant we could not find strong correlations with high fluid volumes. The amount of junctions is supposed to be directly correlated with the number of endpoints and is presumed to be an indicator for angiogenetic activity (Told, Reiter, Schranz, et al., 2020; Told, Reiter, Mittermüller, et al., 2020). However, we did not even find a statistically significant correlation between TNJ and RF at baseline as we did for TNE.

Spaide (2015) evaluated OCTA images of fibrovascular PEDs and concluded that under therapy formation of loops, anastomoses and loss of sprouts reflected maturation of neovascular networks. This finding, if occurring in untreated eyes as a natural maturation process, could explain why the amount of junctions is not necessarily associated with the amount of endpoints, as loops can be formed, and subsequently do not correlate with exudation.

The weak negative correlation between AVL and the amount of fluid can be explained by vessel complexity, and higher AVL indicates less complexity in the form of less junctions and subsequently a reduced number of endpoints.

It cannot be emphasized enough that the low *R*‐squared value of 0.23 indicates that our SRF model, based on these MNV parameters, explains just a small part of the variety within the fluid data. IRF only reached the level of statistical significance with the VA (*p* = 0.04) in a univariable model, but it did not reach the level in a multivariable model. This leads to the assumption that far more factors are involved in the exudation process than the investigated vessel parameters. One hypothesis could be that SRF and IRF might derive from a combination of increased permeability of physiological retinal vessels and RPE dysfunction due to abnormal VEGF levels (Ablonczy et al., 2014). This is supported by the existence of large biologically active (growing in OCTA and ICGA) MNV membranes, which are, however, clinically inactive so non‐exudative. Eyes with these so‐called quiescent MNVs show no signs of fluid, besides vessel formations in OCTA (Carnevali et al., 2018).

Told, Reiter, Schranz, et al., 2020; Told, Reiter, Mittermüller, et al., 2020 reported that a decrease within the MNV parameters such as VA, TVL, JD, TNJ and VD was only detected within the first days after treatment and could not be observed 30 days later. On the contrary, these parameters even increased under treatment over a longer follow‐up period; however, CST stayed stable (Told, Reiter, Schranz, et al., 2020; Told, Reiter, Mittermüller, et al., 2020). Their findings considering the changes in MNV parameters are in line with ours as we did not observe significant changes between baseline and M1.

This is another example supporting the hypothesis that neovascularization and exudation are two findings in the disease process but may not be causally related to each other.

The individual effect of anti‐VEGF treatment on distinct fluid volumes or MNV parameters is well known (Schmidt‐Erfurth et al., 2020; Told, Reiter, Schranz, et al., 2020; Told, Reiter, Mittermüller, et al., 2020); however, the correlations between both and the respond to treatment are still subject to investigation and research.

By analysing correlations between baseline MNV parameters and the corresponding treatment response expressed as fluid decrease measured at M1 and correlations between baseline MNV parameters and absolute fluid in eyes at M1, we tried to shed a different light on this subject.

Since the absolute decrease of RF after anti‐VEGF therapy is dependent on the absolute amount of fluid at baseline, we chose to calculate relative fluid reduction and did not find a statistically significant correlation between MNV vascular parameters and relative fluid decrease, neither in total RF nor in SRF or IRF. This finding suggests that the therapeutic effect of anti‐VEGF agents is independent from the stated MNV OCTA parameters analysed in this study. A significant correlation between the vessel parameters at baseline and the response to treatment was not found.

We found statistically significant but very weak (low R‐squared values) positive correlations for total RF and vessel parameters (VA, VD, TNJ, JD, TVL, AVL and MEL) at M1. MEL was the only parameter which was negatively correlated with the amount of RF, indicating that a inhomogeneous MNV lesion is a sign for less VEGF activity.

Similar results were reported by Zedanly et al. showing that MNV lacunarity was higher (more inhomogeneous) in the so‐called “good responders” (patients having anti‐VEGF treatment every 5 months) compared to “bad responders” (patients having anti‐VEGF treatment every month) (Ozdemir Zeydanli & Gurelik, 2020).

Similar results were observed by analysing vessel parameters and the amount of IRF at this timepoint. VD (*p* < 0.05) and JD (*p* < 0.05) showed positive correlation, MEL (*p* < 0.01) negative, respectively. However, these statistically significant results showed very low correlations (low *R*‐squared values).

We did not find any statistically significant correlations for vessel parameters and SRF at M1.

The FLUID study showed that tolerating some SRF led to non‐inferior visual outcome when compared to patients in which SRF was not tolerated (Guymer et al., 2019). One could assume that this is also the case regarding the correlations between vessel parameters and SRF at M1. However, analogous to the findings by Grechenig et al. (2021), showing a negative effect of SRF on visual acuity the longer the treatment interval is extended, we do not know if this correlation becomes positive in extended treatment intervals.

Recently, Faatz et al. published their results on MNV parameters, such as area, total length of all vessels and fractal dimension, representing complexity of vascular structure, and correlated these parameters with MNV activity presented as central field thickness (CFT). Over a follow‐up period of 9 months, they found that all of the parameters investigated were statistically significantly higher in active MNVs compared to inactive MNVs (Faatz et al., 2019). Even though we analysed distinct fluid volume within the central 6 mm at a certain follow‐up timepoint, we came to the result that MNV activity is only weakly correlated with IRF volume and did not show any correlation with SRF at all for example. This finding substantiates the hypothesis that MNV growth and exudation are due to the same cause but not directly related to each other.

Nevertheless, the effect of changes in MNV parameters on RF over a longer follow‐up period needs further evaluation.

Strengths of this study were a quantitative analysis of the MNV network combined with an AI algorithm for precise quantification of retinal fluid in nanoliters.

The authors are aware of limitations of this study including limits of analysing a 3‐dimensional structure (MNV network) based on a 2‐dimensional slab, and the manual demarcation of the MNV area. Another limitation was the manual adjustment of image process parameters.

However, care was taken to assure the MNV membrane was depicted in its entirety in every single volume scan.

Furthermore, the deep learning fluid quantification algorithm and the export of vol files from the Heidelberg OCT device are not available to every group.

In conclusion, this analysis came to the result that none of the MNV parameters, even though statistically significant, showed moderate or high correlation with any fluid compartment at baseline or after the first anti‐VEGF treatment. The relative fluid decrease after treatment was not correlated at all with any of the investigated parameters. We do not call into question the benefits of the clinical application of OCTA for diagnosis and follow‐up of neovascular AMD, which has been shown previously (Onishi & Fawzi, 2019).

The MNV parameters evaluated in our study, however, did not correlate with a decrease in SRF and IRF and, hence, did not serve as imaging biomarkers for exudative activity in neovascular AMD in our patient cohort.

Subsequently, the presence of fluid itself remains the most important biomarker for treatment decisions. High‐resolution OCT volume scans in addition to automated fluid detection, segmentation and quantification in the form of deep learning networks would improve and facilitate treatment decisions.

## FUNDING INFORMATION

This work was supported by the FWF (Austrian Science Fund; Grant Number KLI 749‐B).

## CONFLICT OF INTERESTS

The authors declare no competing interests.
